# Hepatic population derived from human pluripotent stem cells is effectively increased by selective removal of undifferentiated stem cells using YM155

**DOI:** 10.1186/s13287-017-0517-2

**Published:** 2017-04-17

**Authors:** Seok-Jin Kang, Young-Il Park, So-Ryeon Hwang, Hee Yi, Nga Tham, Hyun-Ok Ku, Jae-Young Song, Hwan-Goo Kang

**Affiliations:** Vet Drugs and Biologics Division, Animal and Plant Quarantine Agency, 177, Hyeoksin 8-ro, Gimcheon-si, Gyeongsangbuk-do 39660 Republic of Korea

**Keywords:** Pluripotent stem cells, Hepatic differentiation, Residual undifferentiated stem cell, YM155

## Abstract

**Background:**

Pluripotent stem cells (PSCs) such as embryonic stem cells and induced pluripotent stem cells are promising target cells for cell regenerative medicine together with recently advanced technology of in-vitro differentiation. However, residual undifferentiated stem cells (USCs) during in-vitro differentiation are considered a potential risk for development of cancer cells and nonspecific lineage cell types. In this study we observed that USCs still exist during hepatic differentiation, consequently resulting in poor quality of the hepatic population and forming teratoma in vivo. Therefore, we hypothesized that effectively removing USCs from in-vitro differentiation could improve the quality of the hepatic population and guarantee safety from risk of teratoma formation.

**Methods:**

Human PSCs were differentiated to hepatocytes via four steps. YM155, a known BIRC5 inhibitor, was applied for removing the residual USCs on the hepatic differentiation. After YM155 treatment, hepatocyte development was evaluated by measuring gene expression, immunostaining and hepatic functions at each stage of differentiation, and forming teratomas were confirmed by cell transplantation with or without YM155.

**Results:**

The selected concentrations of YM155 removed USCs (NANOG^+^ and OCT4^+^) in a dose-dependent manner. As a result, expression of endodermal markers (*SOX17*, *FOXA2* and *CXCR4*) at stage II of differentiation and hepatic markers (*ALB,* AFP and *HNF4A*) at stage III was up-regulated by YM155 treatment as well as the hepatic population (ALB^+^), and functions (ALB/urea secretion and CYP450 enzyme activity) were enhanced at the final stage of differentiation (stage IV). Furthermore, we demonstrated that *NANOG* and *OCT4* expression remaining until stage III (day 15 of differentiation) completely disappeared when treated with YM155 and teratoma formation was effectively prevented by YM155 pretreatment in the in-vitro study.

**Conclusions:**

We suggest that the removal of USCs using YM155 could improve the quantity and quality of induced hepatocytes and eliminate the potential risk of teratoma formation.

**Electronic supplementary material:**

The online version of this article (doi:10.1186/s13287-017-0517-2) contains supplementary material, which is available to authorized users.

## Background

Human pluripotent stem cell (PSC)-induced hepatocytes (iHeps) are a promising target for drug development and cell transplantation. Over the past 10 years, many protocols for hepatocyte induction from embryonic stem cells (ESCs) and induced pluripotent stem cells (iPSCs) have been developed by recapitulating in-vivo hepatic development [1–6]. Acquiring pure hepatocytes is a crucial factor for hepatotoxicity screening of drug candidates and stem cell-derived hepatocyte therapy. Thus far, hepatocyte induction has enabled progress in acquiring highly purified hepatocytes and in enhancing hepatic functions [3, 7–9].

There are many previous reports about undifferentiated stem cells (USCs) that remain during in-vitro differentiation. The residual USCs exist long term in both in-vitro and in-vivo differentiation [10]. This is a considerably critical concern in cell replacement therapy because the residual stem cells have inherent problems (i.e., teratoma formation) [11–13]. Therefore, eliminating the residual USCs is an indispensable step for safer cell therapy and efficient hepatotoxicity screening of drug candidates. Recently, selective removal of USCs has been tried to prevent tumorigenic potential and teratoma formation using antibody-based strategy in clinical fields [14–16]. In addition, small molecules have been suggested to effectively eliminate the risk of teratomas [17, 18]. Of these molecules, YM155 is one of the inhibitors targeting the *BIRC5* (*Survivin*) gene, an anti-apoptotic factor, which is highly overexpressed in ESCs/iPSCs and cancer stem cells. Thus, this molecule can induce selective apoptotic cell death of USCs [18]. Currently, the compound has been introduced in clinical applications for cancer therapy and interruption of teratoma formation after stem cell therapy [19].

In our former study, USCs remained in a small portion during hepatic differentiation and consequently developed nonhepatic phenotypes [20]. Lee et al. [18] reported that YM155 effectively eliminated USCs without affecting differentiated counterparts. However, little research has been conducted to selectively remove the undifferentiated cells during in-vitro hepatic differentiation. In the present study, we investigated the effects of YM155 on hepatic differentiation using human PSCs concerning the effective treatment stage of differentiation and optimal concentration of YM155 and in-vivo teratoma formation. To our knowledge, this is the first report of applying YM155 to in-vitro hepatic differentiation.

## Methods

### Cell culture

Human ESCs (WA01) and iPSCs (iPS(IMR90)-1) were purchased from the WiCell Research Institute (WI, USA; http://wicell.org/). Human iPSCs (QIA7) were established from adipose tissue-derived stromal cells in our laboratory [21]. These cells were mechanically passaged using Dispase (STEMCELL Technologies, Canada) and maintained on a Matrigel-coated plate (BD Bioscience, CA, USA) with mTeSR1 medium (STEMCELL Technologies). HepG2 (ATCC, VA, USA) was cultured in EMEM (ATCC) containing 10% FBS (Gibco-BRL, NY, USA) and 1% penicillin and streptomycin (Millipore, MA, USA). Human cryopreserved primary hepatocytes (p-Heps) were purchased as BD Gentest™ Inducible-Qualified Human CryoHepatocytes (Lot #178; BD Bioscience). Human hepatocytes were cultured in HMM medium using a SingleQuots™ kit (Lonza, MD, USA) on Coning BioCoat Collagen I-coated plates (BD Bioscience). Human adipose tissue-derived stromal cells (hAT-SCs) were maintained with DMEM containing 10% FBS, 50 U/ml penicillin and 50 μg/ml streptomycin.

### Differentiation of PSCs into hepatocytes

For the hepatic differentiation of human PSCs, a previously reported hepatic differentiation protocol designed for human ESCs [1] was applied with some modifications. Briefly, human PSCs were plated at a density of 2.5 × 10^5^ cells/ml on Matrigel-coated six-well plates with mTeSR1 medium (STEMCELL Technologies) including ROCK inhibitor (Y27632; STEMCELL Technologies). The medium was replaced with definitive endodermal induction medium (DE) for 5 days (stage I). The DE medium consisted of RPMI 1640 (without l-glutamine; Gibco-BRL) supplemented with 2 mM l-glutamine (Millipore), 0.5 mg/ml albumin fraction V (Merck Millipore, Germany) and 100 ng/ml Activin A (PeproTech, NJ, USA). Afterward, the definitive endodermal cells were differentiated into hepatoblasts using the hepatic endodermal medium (Hep-1) for 5 days (stage II) followed by hepatic specification medium (Hep-2) for 5 days (stage III). Hep-1 and Hep-2 comprised the HBM SingleQuots™ kit (Lonza) supplemented with 30 ng/ml FGF4 (PeproTech) and 20 ng/ml BMP2 (Invitrogen, MD, USA) at stage II and 20 ng/ml HGF (PeproTech) and 20 ng/ml OSM (R&D Systems, MN, USA) at stage III, respectively. After hepatic specification, the cells were further matured using the HMM SingleQuots™ kit for 5 days (stage IV).

### RT-PCR

Total RNA was isolated from the cells using the RNeasy mini kit (Qiagen, Germany). cDNA was synthesized from 1 μg of total RNA primed with AcuPower cDNA synthesis premix (Bioneer, Republic of Korea). Primer sets are represented in Additional file 1: Table S1. Real-time PCR was performed using the CFX (Bio-Rad, CA, USA) instrument and the SYBR Green script premix (Bio-Rad). Normalization of samples was determined by GAPDH, and all sets of reactions were conducted in triplicate (*n* = 3). The relative expression levels are expressed as a fold-change of the indicated control.

### Immunocytochemistry

For immunostaining, the cells were fixed with 4% paraformaldehyde (PFA; Thermo Scientific) for 5 min after washing with 1× phosphate-buffered saline (PBS). After rinsing with 1× rinse buffer (1× Tris–HCl including 0.05% Tween-20), permeabilization was performed with 0.1% Triton X-100 for 10 min. After blocking with blocking solution (1:20 diluent with 1× PBS) for 30 min at room temperature, each primary antibody was reacted by incubating overnight at 4 °C. After rinsing, secondary antibodies were applied in incubation for 1 h at room temperature. Information for antibodies is listed in Additional file 1: Table S2. The nuclei were stained for 1 min with Hoechst33258 (Invitrogen) diluted in 1× PBS (1:10,000). Immunofluorescence was detected under a fluorescence microscope (Axiovert; Carl Zeiss, Germany).

### Cytotoxicity assay

To measure cytotoxicity by YM155 treatment, QIA7 (stage 0), QIA7-iHeps (stage I and stage II) and hAT-SCs were seeded on Matrigel-coated 96-well plates (2 × 10^4^ cells per well) and cultured for 24 h at 37 °C. Serial concentrations of YM155 (0–100 μM) were diluted with each culture medium as already described and incubated with the cells for 24 h. The culture medium was subsequently replaced with 100 μl of each fresh medium containing 10% (v/v) 2-(4-Iodophenyl)-3-(4-nitrophenyl)-5-(2,4-disulfophenyl)-2H-tetraxolium (WST) reagent (DoGEN, Republic of Korea) and again incubated for 2 h at 37 °C. Absorbance was read using a luminometer (FlexStation III; Molecular Device, CA, USA) at 450 nm, and the results were expressed as the percent of nontreated control.

### Caspase-3 activity

To detect apoptotic cell death, we used the Caspase-3 colorimetric activity assay kit (Millipore). QIA7, QIA7-iHeps (day 7 of differentiation) and hAT-SCs were seeded with a relevant cell number and incubated overnight at 37 °C. Each cell was treated by serial concentrations of YM155 (1–100 nM) for 16 h. After lysis with 1 × 10^6^ cells, the supernatant was incubated with Caspase-3 substrate for 1 h at 37 °C. Absorbance was read using a luminometer (FlexStation III) at 405 nm, and the activities were expressed as a fold-increase against DMSO control.

### Flow cytometry

Cells were dissociated in 0.05% Trypsin–EDTA (Invitrogen) and then resuspended in 10% FBS/DMEM (v/v). The collected cells were fixed with 4% PFA, permeabilized with 0.1% Triton X-100 and stained with an antibody against OCT4 and Albumin. The analyses were performed using FACSCalibur (BD Biosciences).

### Functional assay for hepatocytes

To test hepatic functions with stem cell iHeps at the final day of stage IV (day 20 of differentiation), the differentiated cells were dissociated in Accutase for 30 min and seeded on Collagen I-coated plates with HMM medium. After overnight incubation at 37 °C, each functional test such as periodic acid Schiff (PAS) staining, low-density lipoprotein (LDL) uptake, albumin/urea secretion, CYP450 enzyme activity and drug metabolism was carried out as described in the following. HepG2 and p-Heps were used as control cells.

#### PAS staining

To detect glycogen storage, PAS staining was performed at day 21 of differentiation. The cells were fixed for 5 min with 4% PFA and oxidized for 5 min in periodic acid (Sigma-Aldrich, MO, USA) at room temperature. After rinsing with distilled water for 5 min, the cells were treated with Schiff’s reagent (Sigma-Aldrich) for 15 min.

#### LDL uptake

To estimate uptake of low LDL, LDL-DyLight™ 550 (Abcam) was diluted with culture medium (1:100). Cells were incubated with LDL-DyLight™ 550 working solution for 4 h at 37 °C. The medium was replaced with fresh culture medium. Immunofluorescence was detected under a fluorescence microscope (Axiovert).

#### Albumin and urea secretion

The culture medium was changed at day 21 of differentiation, and the cells were additionally incubated for 24 h at 37 °C. The supernatants collected from each well were centrifuged for 5 min at 3000 × *g* to remove floating cells and stored at –20 °C until assay. The albumin and urea amounts in culture medium were measured using an Albumin Human ELISA kit (Abnova, CA, USA) and urea assay kit (Cell Biolabs, CA, USA), respectively, according to the manufacturer’s instructions. Absorbance was read on a luminometer (FlexStation III) at a wavelength of 450 nm for albumin and 630 nm for urea. The albumin and urea amounts were calculated using each standard curve and normalized by protein concentration (mg/ml).

#### CYP450 enzyme activity

CYP1A2 and CYP3A4 enzyme activities were measured using the CYP450-Glo™ assay kit (Promega, WI, USA) according to the manufacturer’s instructions. The supernatants were removed, and the cells were incubated with substrate (Luciferin-1A2 for CYP1A2 and Luciferin-IPA for CYP3A4) for 1 h. The supernatants of each well were transferred to white opaque 96-well plates. CYP450 activities were then measured using a luminometer (FlexStation III). The results were expressed as a relative activity for control.

#### Drug clearance

To evaluate drug metabolism, 1 μM aflatoxin B1 (Sigma-Aldrich) and 100 μM acetaminophen (Sigma-Aldrich) diluted with HMM medium treated QIA7-iHeps for 24 h, and medium containing test drugs was used as control (no cells). The supernatants were collected, and the concentrations of each compound in the supernatants were determined by HPLC (Waters 2996; Waters, MA, USA). Drug clearance in p-Heps was also performed under the same method. The values were normalized by protein concentration (mg/ml) and expressed by the percentage of control.

### Teratoma formation

For in-vivo cell transplantation, QIA7 and QIA7-iHeps (day 7 of differentiation) pretreated with and without YM155 (5 nM) were dissociated by Dispase and Accutase, respectively. Approximately 1 × 10^6^ cells were prepared in DMEM/F12 (50 μl) and mixed with Matrigel (1:1) on ice. The mixture was injected into the testis of 6-week-old nude mice (BkINbt:BALB/c/nu/nu; NARA-Biotech, Republic of Korea). Six or seven weeks later, the teratomas were dissected. Tumor masses were fixed with 10% neutral buffered formalin (Sigma-Aldrich). Paraffin-embedded tissues were sectioned and stained with hematoxylin and eosin (H&E) and analyzed in the Cell Imaging-Histology core facility at the Quarantine & Inspection Agency.

### Statistical analysis

Results were expressed as the mean ± standard deviation (SD) for triplicate experiments (*n* = 3). The statistical significance was determined using Statistica5.5 (StatSoft, OK, USA) with one-way analysis of variance (ANOVA) and post-hoc comparisons between the control group and each treatment group using Duncan’s multiple comparison test. *p* < 0.05 was considered statistically significant.

## Results

### Hepatic differentiation of human PSCs with the modified protocol

**Fig. 1:**
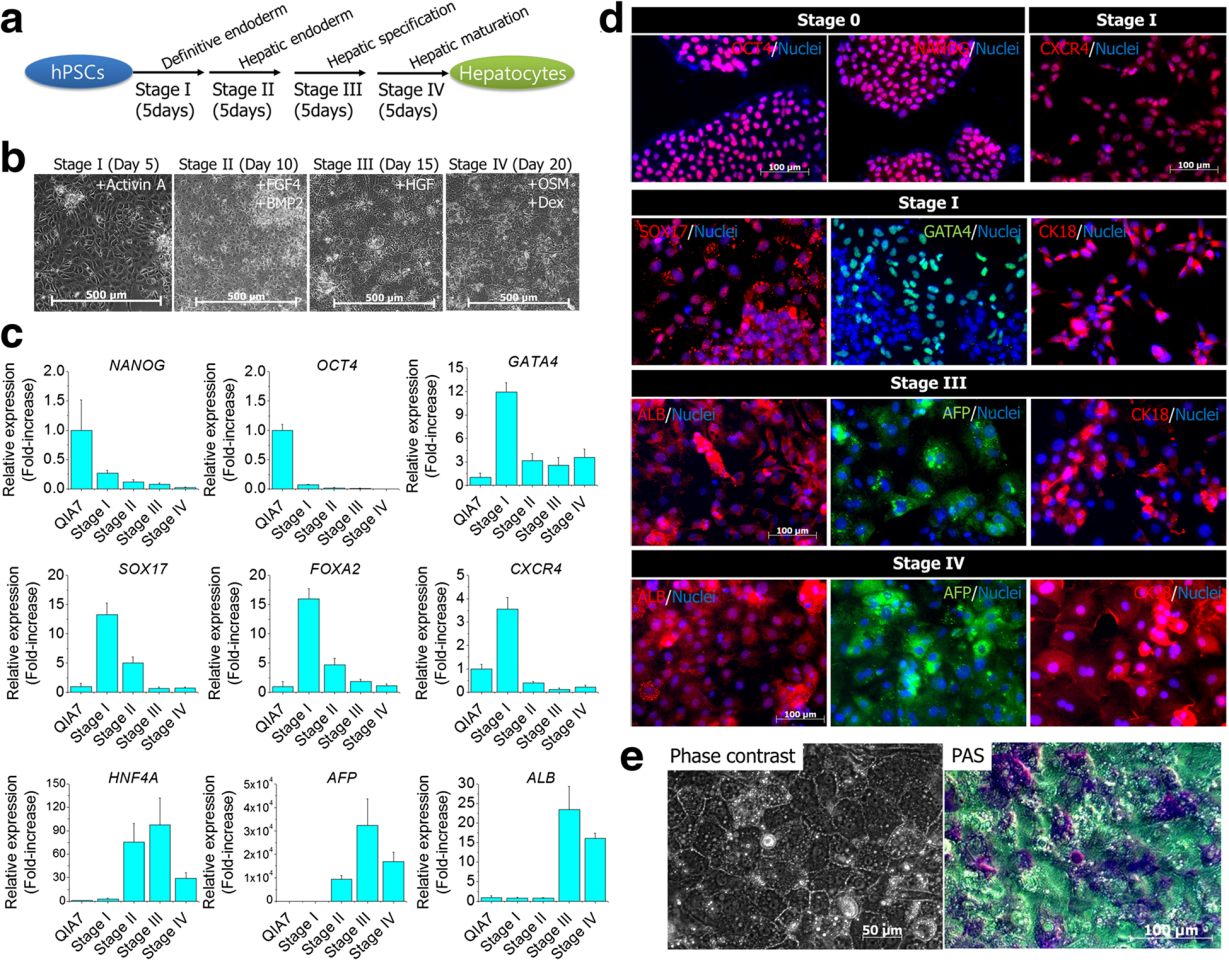
Hepatic differentiation of human PSCs. The modified protocol of hepatic differentiation was distinct at four stages; definitive endoderm, hepatic endoderm, hepatic specification and hepatic maturation (**a**). Under this sequential induction condition, the development of QIA7 to hepatocytes was confirmed at each stage in the aspects of morphological changes (**b**) and expression of stage-specific marker genes (**c**) and proteins (**d**). The differentiated hepatocytes showed a typical hepatocyte-like shapes and glycogen synthesis at stage IV (**e**). *hPSC* human pluripotent stem cell, *AFP* alpha-fetoprotein, *ALB* albumin, *BMP2* bone morphogenetic protein 2, *CK18* cytokeratin 18, *CXCR4* C-X-C chemokine receptor type 4, *FGF4* fibroblast growth factor 4, *FOXA2* forkhead box protein A2, *GATA4*, transcription factor GATA-4, *HGF* hepatocyte growth factor, *HNF4A* hepatocyte nuclear factor 4 alpha, *OCT4* octamer-binding transcription factor, *OSM* oncostatin M, *PAS* periodic acid Schiff, *SOX17* sex determining region Y-Box 17

To achieve hepatic differentiation, human PSCs were differentiated to hepatocytes for 20 days via four sequential stages; definitive endoderm (5 days), hepatic endoderm (5 days), hepatic specification (5 days) and hepatic maturation (5 days) (Fig. 1a). Human PSCs were induced to definitive endoderm using Activin A (100 ng/ml) for 5 days. During the whole induction period of definitive endoderm, a large number of differentiated cells was easily detached from the plate. Thus, the relevant cell number was needed for protocol optimization. In our preliminary results, the optimal density of cells was approximately 2× 10^4^–3 × 10^4^ cells/cm^2^ in human PSCs (data not shown). Under this condition, QIA7 showed stage-specific morphology at each stage (Fig. 1b). For expression of stage-specific marker genes shown in Fig. 1c, pluripotent genes such as *NANOG* and *OCT4* rapidly decreased at stage I. On the contrary, *GATA4*, *SOX17*, *FOXA2* and *CXCR4* as definitive endodermal markers were highly expressed at stage I, and expression diminished thereafter. For the hepatic markers, expression of *HNF4A* and *AFP* started to be increased at stage II, and expression was maximized at stage III. *ALB* was not detected at stage II, and expression was sharply enhanced at stage III. However, three hepatic marker genes were decreased at stage IV. The expression patterns of genes were similar to those of marker proteins (Fig. 1d and Additional file 2: Figure S1). Cytokeratin 18 (CK18)-positive cells were detected during the whole period of differentiation. QIA7-iHeps showed a typical hepatocyte phenotype and glycogen storage at the final stage (Fig. 1e).

### Characterization of nonhepatic lineage cells derived from USCs

**Fig. 2:**
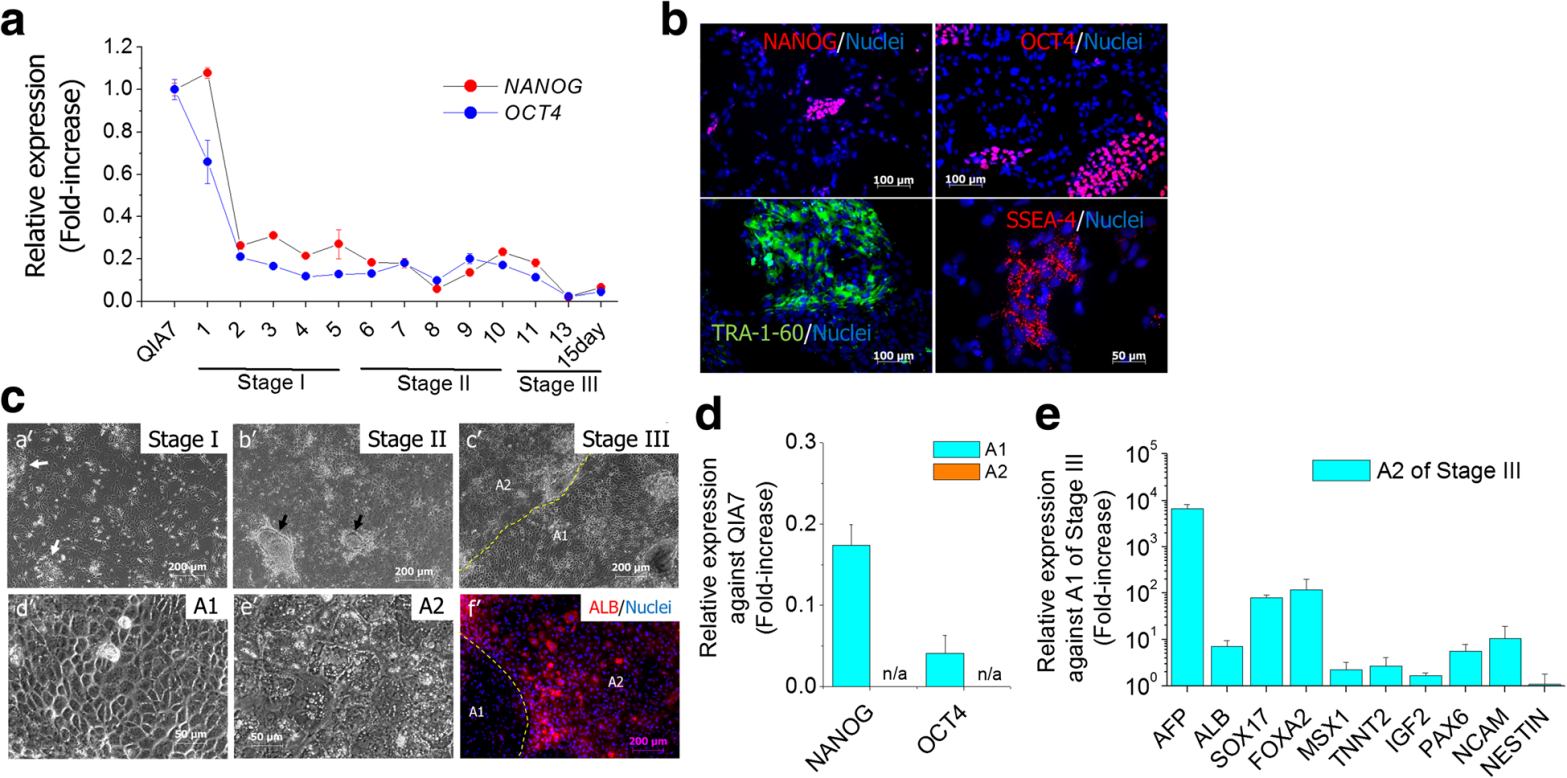
Characterization of nonhepatic lineage cells derived from USCs during hepatic differentiation. Expression of *NANOG* and *OCT4* as pluripotent genes was traced to confirm the presence of residual USCs during hepatic differentiation (**a**). Additionally, human pluripotent stem cell-specific markers such as NANOG, OCT4, TRA-1-60 and SSEA-4 were detected in cell clusters at day 5 of hepatic differentiation (stage I) (**b**). (**c**) The cell clusters (*arrow*) of stage I (*a*') were tightly aggregated during stage II (*arrow*) (*b*'). Thereafter, the aggregated cells rapidly expanded to nonhepatic lineage cells (area 1 (*A1*)) distinguishable from typical hepatic phenotype cells (area 2 (*A2*)) at stage III (*c*'). (*d*', *e*') Magnification of A1 and A2 in (*c*'), respectively. A1 and A2 were stained with Albumin antibody (*f*'). Nuclei were stained with Hoechst33258. For further characterization, expression of pluripotent genes (*NANOG* and *OCT4*) (**d**) and lineage-specific genes (*AFP*, *ALB*, *SOX17*, *MSX1*, *TNNT2*, *IGF2*, *PAX6*, *NCAM* and *NESTIN*) (**e**) was compared between A1 and A2 at stage III. *AFP* alpha-fetoprotein, *ALB* albumin, *FOXA2* forkhead box protein A2, *IGF* insulin growth factor2,* ﻿MSX1* msh homeobox-1,﻿ *NCAM* neural cell adhesion molecule, *OCT4* octamer-binding transcription factor, *PAX6* paired box protein Pax﻿-6﻿﻿, *SOX17* sex determining region Y-Box 17, *TNNT2* cardiac muscle troponin T

To characterize the residual USCs, pluripotent genes and marker proteins were investigated under our modified protocol of hepatic differentiation. The expression of *NANOG* and *OCT4* did not disappear until day 15 of differentiation (stage III), although the expression sharply declined by day 2 of differentiation (Fig. 2a). Most of the clusters still remaining at stage I (day 5 of differentiation) expressed pluripotent markers such as NANOG, OCT4, TRA-1-60 and SSEA-4 (Fig. 2b). Thus, we hypothesized that the USCs caused cellular heterogeneity in the hepatic differentiation. QIA7 actively differentiated to definitive endodermal cells after exposure to Activin A at stage I. However, many cell clusters still expressing the pluripotent markers were observed on the surrounding edges of definitive endodermal cells at stage I (Fig. 2c, a'). The clusters were tightly aggregated as soon as BMP2 and FGF4 were treated at stage II (Fig. 2c, b'). Thereafter, the cell population derived from the aggregated cells rapidly expanded at stage III (Fig. 2c, c'). This cell population (Fig. 2c, d', magnified in area 1 (A1) of Fig. 2c, c') accounted for the majority of the cell population at the final stage of differentiation and distinguishable from typical hepatic phenotype in area 2 (A2) of Fig. 2c, c' (Fig. 2c, e'). During immunostaining, the cells of A1 were negative for ALB, but most of the cells in A2 were positive (Fig. 2c, f'). To further characterize A1 and A2, each cell population was separately collected according to distinct cell morphology. Interestingly, expression of *NANOG* and *OCT4* was still present in A1 whereas it was not detected in A2 (Fig. 2d). As compared with A1, A2 highly expressed endodermal marker genes (*AFP*, *ALB*, *SOX17* and *FOXA2*) (Fig. 2e).

### Effects of YM155 on hepatic differentiation

**Fig. 3:**
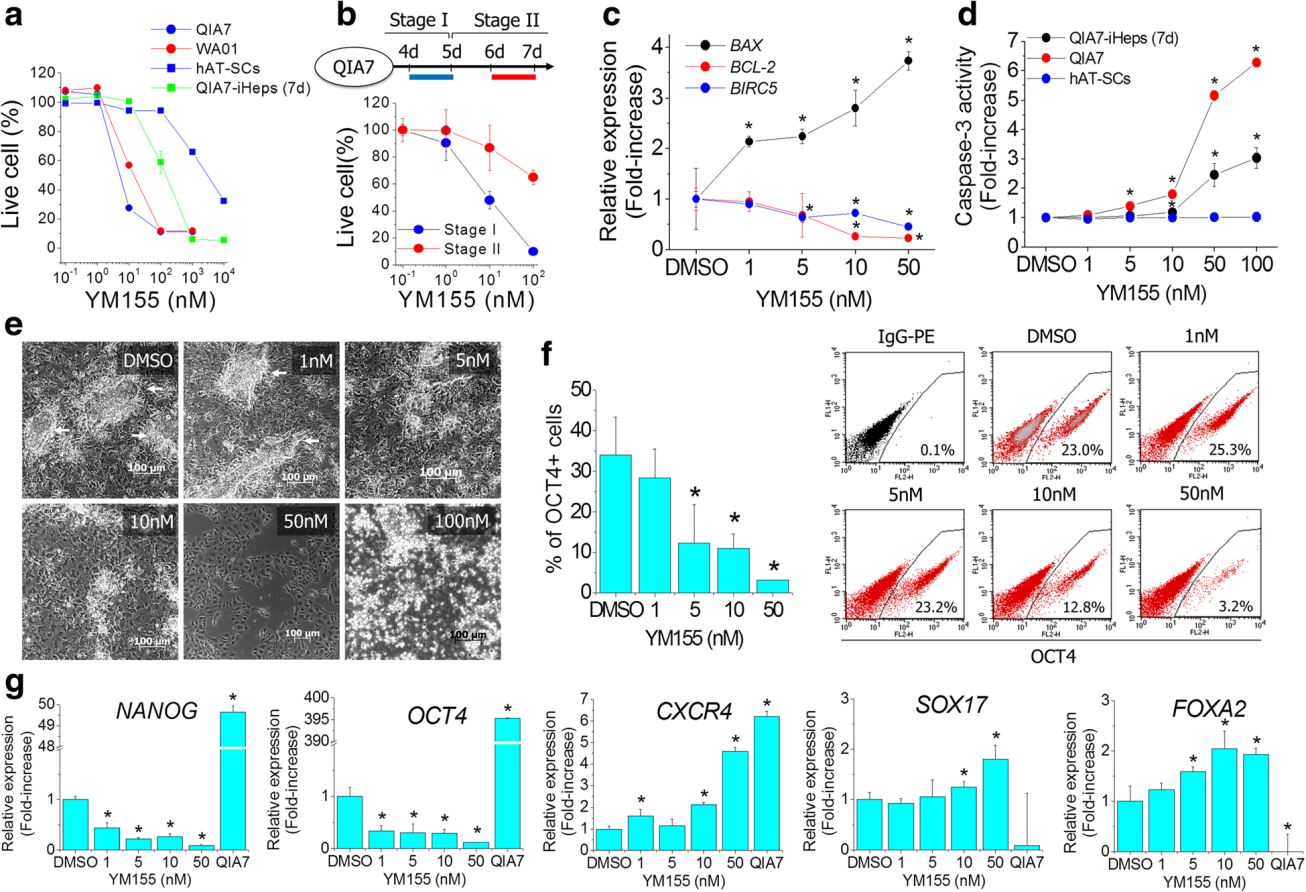
Removal of residual USCs using YM155. Cytotoxicity was measured by WST after QIA7, WA01, QIA7-iHeps and hAT-SCs were treated with YM155 (0–10,000 nM) for 24 h (**a**). With the selected concentrations (from 1 to 100 nM), cell viability was estimated at stage I of QIA7 (day 4) and stage II (day 6) (**b**). Expression of apoptosis-related genes (*BIRC5*, *BCL-2* and *BAX*) was measured after 24 h of YM155 treatment at stage II (**c**). To further evaluate YM155-induced apoptotic cell death, Caspase-3 activity was estimated after 16-h incubation with YM155 in QIA7, QIA7-iHeps and hAT-SCs (**d**). YM155 removed the cell clusters (*arrow*) in a concentration-dependent manner (**e**). The decrease of USCs was confirmed by flow cytometer using OCT4 antibody (**f**). Pluripotent genes (*NANOG* and *OCT4*) were decreased by YM155, but endodermal genes (*CXCR4*, *SOX17* and *FOXA2*) were increased in a dose-dependent manner (**g**).*p < 0.05 (significantly different from the control). *BAX* bcl-2-like protein 4, *BCL-2* B-cell lymphoma 2, *BIRC5* baculoviral inhibitor of apoptosis repeat-containing 5, *CXCR4* C-X-C chemokine receptor type 4, *DMSO* dimethyl sulfoxide, *FOXA2* forkhead box protein A2, *hAT-SC* human adipose tissue-derived stromal cell, *iHep* induced hepatocyte, *OCT4* octamer-binding transcription factor, *SOX17* sex determining region Y-Box 17

We hypothesized that highly qualified and quantified hepatocytes could be obtained if USCs are effectively removed during hepatic differentiation. To perform selective removal of USCs, YM155 was used in this study. Firstly, serial concentrations of YM155 were used to treat human PSCs (QIA7 and WA01), differentiating cells (QIA7-iHeps) and hAT-SCs as a somatic cell for determining the relevant treatment concentration (Fig. 3a). Each cell type showed different dose responses against YM155 (Fig. 3a). The inhibitory concentration 50 (IC_50_) of QIA7, WA01, QIA7-iHeps and hAT-SCs was 8 nM, 14 nM, 130 nM and 3086 nM, respectively. Human PSCs were affected by lower concentrations of YM155 than hAT-SCs and QIA-iHeps. Therefore, we selected the ranges of YM155 (1–100 nM) for later study. To determine an optimal time point of YM155 treatment, the selected concentrations of YM155 were treated at stage I and stage II. In our preliminary study, the more YM155 treatment at stage I, the more cells survived (Additional file 2: Figure S2). Most of the differentiating cells were easily detached from plates by YM155 treatment at stage I. Therefore, YM155 was not applicable at stage I. On the contrary, the differentiating cells were more tolerant to YM155 treatment at stage II (day 6 of differentiation) than at stage I (day 4 of differentiation) (Fig. 3b). To investigate cytotoxic effects of YM155 at stage II, we estimated changes of apoptosis-related genes on the whole differentiation (Additional file 2: Figure S3). *BIRC5* was down-regulated from day 1 of differentiation and maintained during the whole hepatic differentiation. *BAX*, a pro-apoptotic gene, was up-regulated from stage II to stage III. The expression of *BCL-2*, an anti-apoptotic gene, was not detectable or was at a very low level (data not shown). After YM155 treatment at stage II (day 6 of differentiation), *BIRC5* and *BCL-2* were dose-dependently decreased as compared with DMSO whereas *BAX* was increased (Fig. 3c). Likewise, Caspase-3 activity was significantly increased by a lower concentration of YM155 in QIA7 more than QIA7-iHeps but was not affected in hAT-SCs (Fig. 3d). These results were very similar with Annexin V staining (Additional file 2: Figure S4). In the change of cell phenotype, cell clusters were effectively removed by more than 5 nM of YM155 but not by 1 nM and DMSO (Fig. 3e). However, all cells were dead with 100 nM of YM155 treatment. The cells affected by YM155 were closely consistent with TRA-1-60^+^ cells (Additional file 2: Figure S5). Likewise, OCT4^+^ cells were significantly decreased by more than 5 nM of YM155 in FACS analysis (Fig. 3f). In the genetic analysis, expression of *NANOG* and *OCT4* was decreased and expression of endodermal marker genes such as *CXCR4*, *SOX17* and *FOXA2* was increased in a dose-dependent manner (Fig. 3g). Taken together, the residual USCs were effectively eliminated by YM155 at stage II (day 6 of differentiation) as compared with differentiating cells and somatic cells.

### Evaluation of hepatic induction followingYM155 treatment at stage II

**Fig. 4:**
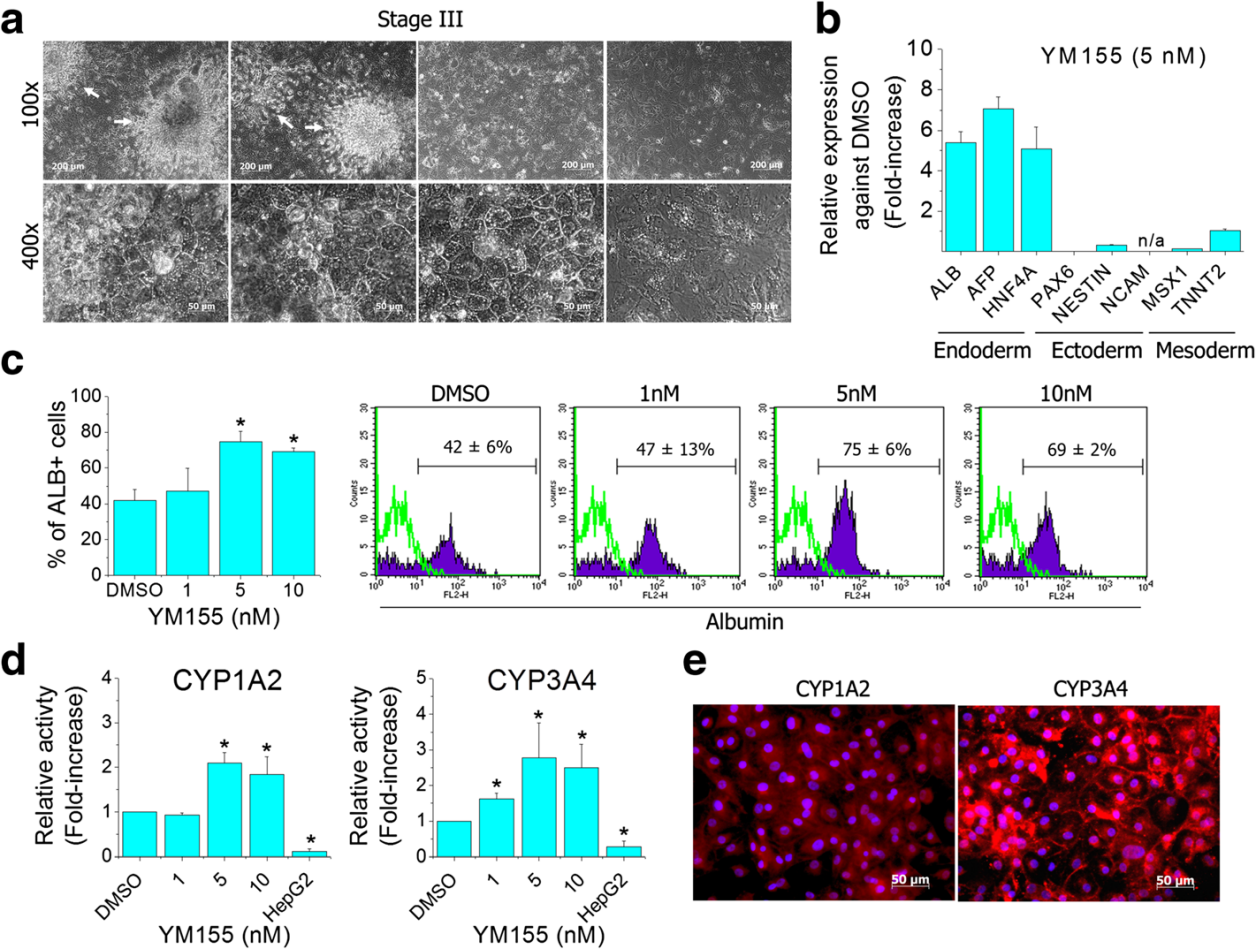
Characterization of hepatic induction following YM155 treatment at stage II. At stage III, the homogeneity of the cell population was observed in 5 and 10 nM YM155 treatment of stage II (day 6), but nonhepatocyte-like cells (*arrow*) derived from USCs still appeared in 1 nM YM155 like DMSO (**a**). Expression of hepatic endoderm (*AFP*, *ALB* and *HNF4A*), ectoderm (*PAX6*, *NESTIN* and *NCAM*) and mesoderm (*MSX1* and *TNNT2*) marker genes was estimated at 15 days of differentiation (**b**). At stage IV, albumin-positive cells were measured by flow cytometer (*n* = 3) (**c**) and enzyme activities of CYP1A2 and CYP3A4 were also estimated with QIA7-iHeps (**d**). QIA7-iHeps showed weak immunoreactivity against CYP1A2 but strong against CYP3A4 (**e**). *p < 0.05 (significantly different from the control). *AFP* alpha-fetoprotein, *ALB* albumin, *DMSO* dimethyl sulfoxide, *HNF4A* hepatocyte nuclear factor 4 alpha, *PAX6* paired box protein Pax-6, *MSX1* Msh homeobox-1, *NCAM* neural cell adhesion molecule, *TNNT2* cardiac muscle troponin T

The level of hepatic differentiation was evaluated at stage III (day 15 of differentiation) after YM155 treatment for 24 h at stage II (day 6 of differentiation). Cellular homogeneity of differentiated cells was observed by 5 nM of YM155 compared with DMSO and 1 nM of YM155 (Fig. 4a). In the group treated with 5 nM of YM155, expression of hepatic endodermal genes *ALB*, *AFP* and *HNF4A* increased significantly in the differentiated cells compared with DMSO. On the contrary, expression of *PAX6*, *NESTIN* and *NCAM* for ectodermal markers and *MSX1* and *TNNT2* for mesodermal genes was decreased in the cells (Fig. 4b). To estimate the purity of hepatocytes in each population, FACS analysis was conducted by immunostaining ALB antibody. Albumin-positive cells increased approximately 1.8-fold with 5 nM of YM155 (75 ± 6%) and 1.6-fold with 10 nM (69 ± 2%) compared with DMSO (42 ± 6%) (Fig. 4c). In a further functional assay conducted on QIA7-iHeps pretreated with YM155 at stage II, activities of CYP450 enzymes showed significant increases in 5 nM by approximately 2.1-fold for CYP1A2 and 2.8–fold for CYP3A4 (Fig. 4d). In immunocytochemical staining, however, QIA7-iHeps showed weak immunoreactivity against CYP1A2 but strong immunoreactivity for CYP3A4 (Fig. 4e).

**Fig. 5:**
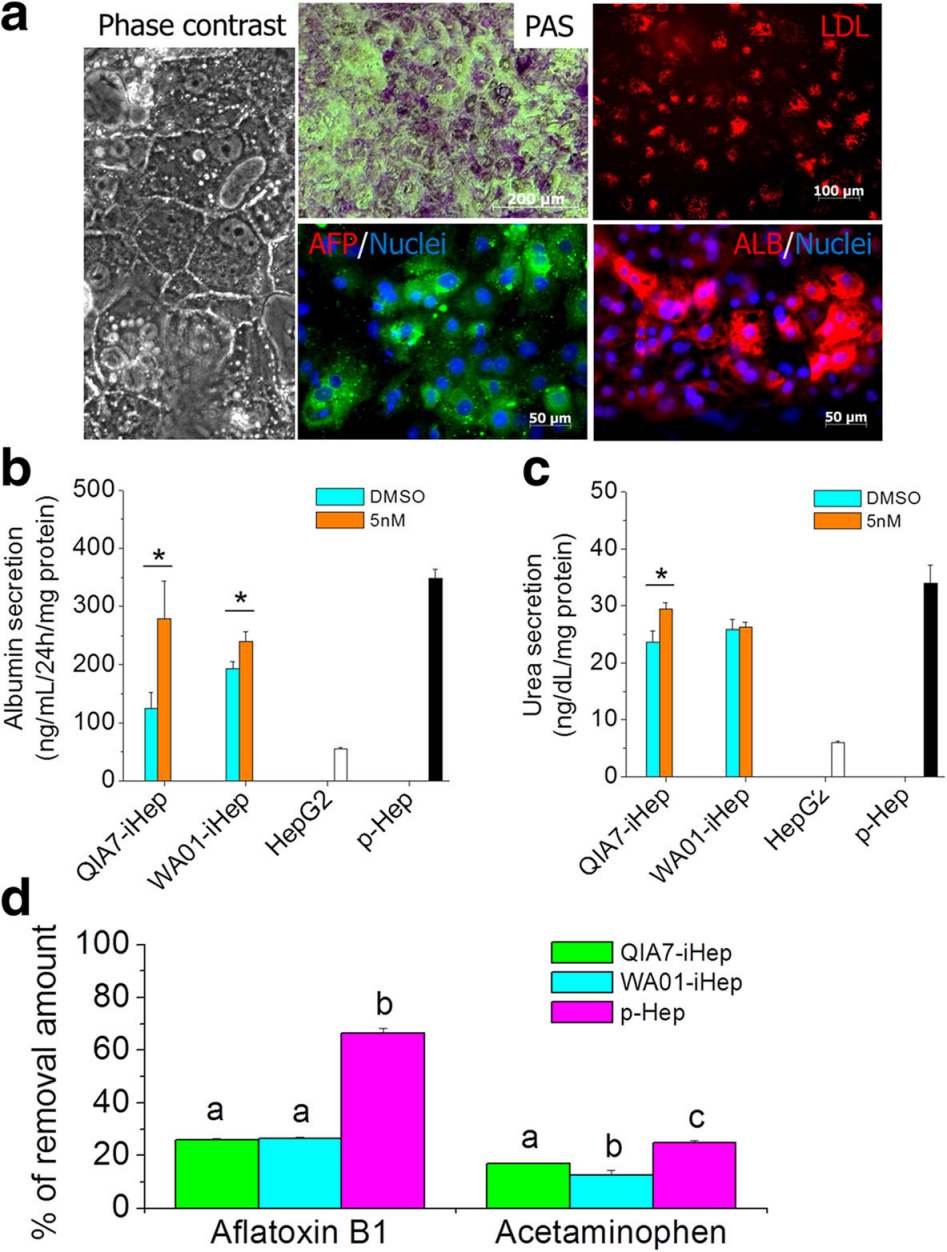
Hepatic functional tests with stem cell iHeps pretreated withYM155 at stage II. WA01-iHeps pretreated with 5 nM YM155 at stage II showed the typical hepatic morphology, glycogen synthesis, LDL uptake and immunoreactivity of AFP and ALB (**a**). With stem cell iHeps (QIA7-iHeps and WA01-iHeps) pretreated by 5 nM YM155 selected as the optimal concentration at stage II, the amount of albumin (**b**) and urea (**c**) secreted for 24 h at day 22 of differentiation was measured and compared with DMSO control, HepG2 and p-Heps. Also, drug clearance was determined with aflatoxin B1 (1 μM) and acetaminophen (100 μM) (**d**). After each compound was incubated with QIA7-iHeps, WA01-iHeps and p-Heps for 24 h, the concentration of each compound was measured using the HPLC method (**d**). *p < 0.05 and different superscript letters indicate significant differences. *AFP* alpha-fetoprotein, *ALB* albumin, *DMSO* dimethyl sulfoxide, *iHep* induced hepatocyte, *LDL* low density lipoprotein, *PAS* periodic acid Schiff

Collectively, 5 nM of YM155 at stage II was chosen as the optimal concentration and time point to generate QIA7-iHeps in an aspect of cell purity and function. With 5 nM of YM155 at stage II, WA01-iHeps differentiated from hESCs showed typical characteristics of hepatocytes, cuboidal morphology and glycogen synthesis (PAS staining), LDL uptake and immunoreactivity against AFP and ALB (Fig. 5a). Further validation was conducted with 5 nM of YM155 in QIA7-iHeps and WA01-iHeps and compared with control cells (HepG2 and p-Heps). The amounts of albumin increased over approximately 2.2-fold in QIA7-iHeps and 1.3-fold in WA01-iHeps (Fig. 5b). In urea secretion, the increases showed 1.3-fold in QIA7-iHeps and no significant difference in WA01-iHeps (Fig. 5c). Even though the tested hepatic functions were improved by YM155 treatment (5 nM) and showed a similar level with p-Heps, the clearance ratios of tested compounds in QIA7-iHeps and WA01-iHeps were significantly lower than those of p-Heps; aflatoxin B1 (25.9%, 26.4% and 66.4%, respectively) and acetaminophen (17%, 12.6% and 24.9%, respectively) (Fig. 5d).

### YM155 induces selective cell death of USCs and prevents teratoma formation

**Fig. 6:**
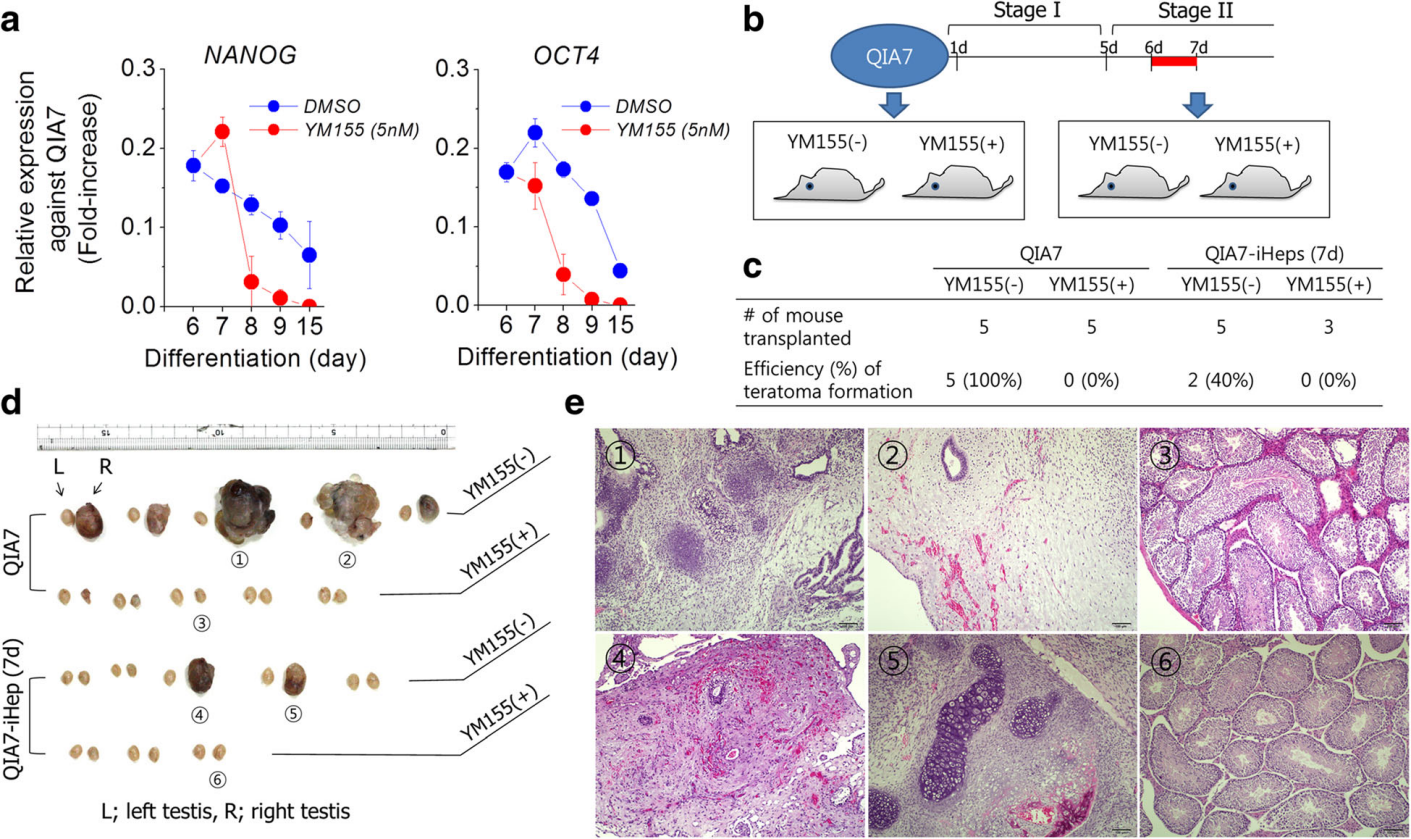
Prevention of a potential risk of USCs using YM155 in vitro and in vivo. Expression of pluripotent genes (*NANOG* and *OCT4*) was traced from days 6 to 15 with the group of 5 nM YM155 pretreated at stage II (**a**). For teratoma formation, QIA7 (stage 0) and QIA7-iHeps (stage II) were pretreated with or without YM155 for 24 h and approximately 1 × 10^6^ cells were injected into the right testis of all mouse (**b**). After 6–7 weeks, teratoma forming was estimated and compared among the groups (**c**, **d**). Testes indicated by numbers in (**d**) underwent histological analysis (**e**). Teratoma mass derived from QIA7 (*①* and *②*) and QIA7-iHeps (*④* and *⑤*) without YM155 treatment showed tissues of three germlayers but testes injected with YM155-pretreated donor cells showed a typical testis structure(*③* and *⑥*). *iHep* induced hepatocyte, *OCT4* octamer-binding transcription factor

Based on our in-vitro results, we tested whether YM155 treatment could prevent teratoma formation in vivo after cell transplantation. Firstly, we confirmed that expression of pluripotent marker genes, such as *NANOG* and *OCT4*, was rapidly decreased after 5 nM of YM155 treatment compared with control, and the two genes completely disappeared at day 15 of differentiation (Fig. 6a). For cell transplantation, we prepared QIA7 and QIA7-iHeps (day 7 of differentiation) pretreated with (YM155^+^) or without 5 nM of YM155 (YM155^–^) for 24 h, respectively (Fig. 6b). As shown in Fig. 6c, d, all mouse testes (*n* = 5) injected with QIA7 (YM155^–^) developed teratomas after 6–7 weeks whereas the counterparts (*n* = 5) injected with QIA7 (YM155^+^) did not form any teratoma-like tumor mass. Interestingly, we observed that QIA7-iHeps (YM155^–^) could develop tumor mass (two of five testes; 40%) but not in YM155 pretreatment (YM155^+^). In the histological analysis, these teratomas showed tissues of all three germlayers (Fig. 6e and Additional file 2: Figure S6).

## Discussion

In the present study, we confirmed that human PSCs differentiated into hepatocyte-like cells via four sequential steps modified from the protocol of Cai et al. [1] by demonstrating the expression of hepatic genes, such as *HNF4A*, *AFP* and *ALB*, and functional hepatic proteins, such as ALB and AFP. Contrary to our expectation, expression of *ALB* and *HNF4A* decreased at stage IV. This might be derived from a nonoptimal culture condition of stage IV (no evaluation in this study) or a relatively low ratio of hepatic purity due to expansion of nonhepatic lineage cells. Morphologically nonhepatic cells (A1) were actually observed at stage III and expanded rapidly during the differentiation. This cell population (A1) showed a distinct morphology against hepatic population as a counterpart (A2) and also contained USCs (Fig. 2d). Additionally, those cells were negative for albumin protein (mature hepatocyte marker), overexpressed *AFP* and *IGF2* (fetal hepatic genes) and expressed mesoderm and ectoderm origin genes. These findings suggest that these cell clusters were derived from USCs and finally reduced cellular homogeneity at the final stage of hepatic differentiation. Therefore, the efficiency of hepatic differentiation could be improved when USCs are selectively eliminated during differentiation.

Small molecules target anti-apoptotic factors to remove residual USCs [17, 18, 22]. Of these small molecules, YM155 in particular triggers apoptosis of USCs but not that of their differentiated derivatives [18]. Likewise, we also demonstrated that human PSCs were uniquely affected by YM155 within the specific ranges (1–10 nM) more than differentiating cells and somatic cells (Fig. 3a). However, it was difficult to apply YM155 at stage I because almost all cells were easily detached from the plate. Therefore, YM155 was applied at stage II. After YM155 treatment, we confirmed apoptotic cell death by apoptotic gene expression, Caspase-3 activity and Annexin V staining. This apoptosis was defined in USCs positive against TRA-1-60 and OCT4. Taken together, YM155 effectively removed USCs at stage II of the differentiation procedure. Finally, this selective elimination resulted in enhancing the expression of endodermal marker genes (*CXCR4*, *SOX17* and *FOXA2*) at stage II and hepatic marker genes (*ALB*, *AFP* and *HNF4A*). We think these increases might be compensation following the decrease of nonspecific lineages.

Drug metabolization is one of the key functions of the liver. The role is primarily conducted by phase I enzymes (CYP450 enzymes) during xenobiotic exposure. Here, the activity of CYP1A2 and CYP3A4 in QIA7-iHeps was significantly increased by 5 nM of YM155 compared with no treatment due to the decrease of the nonhepatic population. However, drug clearance of QIA7-iHeps and WA01-iHeps was significantly lower than that of p-Heps. Aflatoxin B1 and acetaminophen used in this study were metabolized mainly by CYP1A2 and CYP3A4 in hepatocytes. In our previous study, the activity of CYP1A2 and CYP3A4 in QIA7-iHeps and WA01-iHeps was approximately half that of primary hepatocytes [20]. The data are considerably correlated with drug clearance in this study. These lower metabolic activities in QIA7-iHeps and WA01-iHeps compared with p-Heps might be derived from the individual variation of CYP450 genes (i.e., single nucleotide polymorphism) and/or levels of hepatic purity and maturation. A combination of YM155 and additional factors [5, 7, 23, 24] as well as a 3D culture system [8, 25, 26] will be able to demonstrate these issues.

We confirmed that pluripotent genes (*NANOG* and *OCT4*) were completely down-regulated after YM155 treatment and were nearly prevented at the final day of stage II. Finally, expression was not detected at 15 day of differentiation. Under our optimized concentration of YM155 at stage II, USCs could efficiently induce apoptotic cell death. Also, YM155 effectively prevented teratoma formation in our in-vivo study. Interestingly, teratoma occurrence was observed in the transplantation of QIA7-iHeps without YM155. This might be derived from residual USCs on differentiation. Residual USCs accounted for approximately 20–30% of the whole differentiating cells based on the expression of pluripotent marker genes and proteins at stage II. Arithmetically, we can presume that approximately 2 × 10^5^ cells remaining as USCs were injected into the testis. Finally, these cells induced teratoma (40%). Based on a previous study [27], this cell number is sufficient to induce teratoma formation. On the contrary, QIA7-iHeps pretreated with YM155 did not form anything. Regarding cell therapy, the elimination of residual USCs is one of the major concerns to guarantee safety. Based on our evidence, we will be able to utilize the differentiated cells safely as a donor in cell transplantation.

## Conclusions

YM155 could selectively eliminate USCs with an effective treatment time point and optimal concentration on hepatic differentiation. The removal of residual USCs enhanced the efficiency of hepatic differentiation as well as reduced a potential risk of teratoma formation. To our knowledge, this is the first report to demonstrate the effects of YM155 on in-vitro hepatic differentiation. Based on recently advanced technology of hepatic differentiation, application of YM155 will allow generation of a pure hepatocyte population and safety of cell transplantation in regenerative medicine.

## Additional files

Additional file 1: is **Table S1.** presenting a list of primer sets and **Table S2.** presenting antibody information. (DOCX 31 kb)

Additional file 2: contains supplementary materials and methods presenting TRA-1-60 livestaining and Annexin V staining; **Figure S1.** showing the immunocytochemical phenotype stage on hepatic differentiation; **Figure S2.** showing the YM155 effect on stage I; **Figure S3.** showing expression of apoptosis-related genes (*BIRC5* and *BAX*) during the whole hepatic differentiation of QIA7; **Figure S4.** showing Annexin V staining with QIA7, QIA7-iHeps (7 days of differentiation) and hAT-SCs after YM155 treatment for 24 h; **Figure S5.** showing the livestaining of TRA-1-60 at stage II (day 7 of differentiation) after YM155 treatment for 24 h; and **Figure S6.** showing the histological analysis of teratomas derived from QIA7 and QIA7-iHeps without pretreatment of YM155. (DOCX 11505 kb)

## Abbreviations

AFP: Alpha-fetoprotein
ALB: Albumin
BAX: Bcl-2-like protein 4
BCL-2: B-cell lymphoma 2
BIRC5: Baculoviral inhibitor of apoptosis repeat-containing 5
BMP2: Bone morphogenetic protein2
CK18: Cytokeratin 18
CXCR4: C-X-C chemokine receptor type 4
CYP450: Cytochrome P450
DMEM: Dulbecco’s Modified Eagle Medium
DMSO: Dimethyl sulfoxide
EDTA: Ethylenediaminetetraacetic acid
EMEM: Eagle’s minimum essential medium
FBS: Fetal bovine serum
FGF4: Fibroblast growth factor 4
FOXA2: Forkhead box protein A2
GAPDH: Glyceraldehyde 3-phosphate dehydrogenase
GATA4: Transcription factor GATA-4
HGF: Hepatocyte growth factor
HNF4A: Hepatocyte nuclear factor 4 alpha
IGF2: Insulin growth factor2
MSX1: Msh homeobox-1
NCAM: Neural cell adhesion molecule
OCT4: Octamer-binding transcription factor
OSM: Oncostatin M
PAS: Periodic acid Schiff
PAX6: Paired box protein Pax-6
ROCK: Rho-associated protein kinase
SOX17: Sex determining region Y-Box 17
TNNT2: Cardiac muscle troponin T

## References

[1] Cai J, Zhao Y, Liu Y, Ye F, Song Z, Qin H (2007). Directed differentiation of human embryonic stem cells into functional hepatic cells. Hepatology.

[2] Yamamoto H, Quinn G, Asari A, Yamanokuchi H, Teratani T, Terada M (2003). Differentiation of embryonic stem cells into hepatocytes: biological functions and therapeutic application. Hepatology.

[3] Hay DC, Zhao D, Fletcher J, Hewitt ZA, McLean D, Urruticoechea-Uriguen A (2008). Efficient differentiation of hepatocytes from human embryonic stem cells exhibiting markers recapitulating liver development in vivo. Stem Cells.

[4] Si-Tayeb K, Noto FK, Nagaoka M, Li J, Battle MA, Duris C (2010). Highly efficient generation of human hepatocyte-like cells from induced pluripotent stem cells. Hepatology.

[5] Touboul T, Hannan NR, Corbineau S, Martinez A, Martinet C, Branchereau S (2010). Generation of functional hepatocytes from human embryonic stem cells under chemically defined conditions that recapitulate liver development. Hepatology.

[6] Shan J, Schwartz RE, Ross NT, Logan DJ, Thomas D, Duncan SA (2013). Identification of small molecules for human hepatocyte expansion and iPS differentiation. Nat Chem Biol.

[7] Bone HK, Nelson AS, Goldring CE, Tosh D, Welham MJ (2011). A novel chemically directed route for the generation of definitive endoderm from human embryonic stem cells based on inhibition of GSK-3. J Cell Sci.

[8] Hay DC (2013). Rapid and scalable human stem cell differentiation: now in 3D. Stem Cells Dev.

[9] Asplund A, Pradip A, van Giezen M, Aspegren A, Choukair H, Rehnstrom M (2016). One standardized differentiation procedure robustly generates homogenous hepatocyte cultures displaying metabolic diversity from a large panel of human pluripotent stem cells. Stem Cell Rev.

[10] Fu W, Wang SJ, Zhou GD, Liu W, Cao Y, Zhang WJ (2012). Residual undifferentiated cells during differentiation of induced pluripotent stem cells in vitro and in vivo. Stem Cells Dev.

[11] Fujikawa T, Oh SH, Pi L, Hatch HM, Shupe T, Petersen BE (2005). Teratoma formation leads to failure of treatment for type I diabetes using embryonic stem cell-derived insulin-producing cells. Am J Pathol.

[12] Teramoto K, Hara Y, Kumashiro Y, Chinzei R, Tanaka Y, Shimizu-Saito K (2005). Teratoma formation and hepatocyte differentiation in mouse liver transplanted with mouse embryonic stem cell-derived embryoid bodies. Transplant Proc.

[13] Miura K, Okada Y, Aoi T, Okada A, Takahashi K, Okita K (2009). Variation in the safety of induced pluripotent stem cell lines. Nat Biotechnol.

[14] Naujok O, Kaldrack J, Taivankhuu T, Jorns A, Lenzen S (2010). Selective removal of undifferentiated embryonic stem cells from differentiation cultures through HSV1 thymidine kinase and ganciclovir treatment. Stem Cell Rev.

[15] Schriebl K, Satianegara G, Hwang A, Tan HL, Fong WJ, Yang HH (2012). Selective removal of undifferentiated human embryonic stem cells using magnetic activated cell sorting followed by a cytotoxic antibody. Tissue Eng Part A.

[16] Ben-David U, Gan QF, Golan-Lev T, Arora P, Yanuka O, Oren YS (2013). Selective elimination of human pluripotent stem cells by an oleate synthesis inhibitor discovered in a high-throughput screen. Cell Stem Cell.

[17] Bieberich E, Silva J, Wang G, Krishnamurthy K, Condie BG (2004). Selective apoptosis of pluripotent mouse and human stem cells by novel ceramide analogues prevents teratoma formation and enriches for neural precursors in ES cell-derived neural transplants. J Cell Biol.

[18] Lee MO, Moon SH, Jeong HC, Yi JY, Lee TH, Shim SH (2013). Inhibition of pluripotent stem cell-derived teratoma formation by small molecules. Proc Natl Acad Sci U S A.

[19] Rauch A, Hennig D, Schafer C, Wirth M, Marx C, Heinzel T (2014). Survivin and YM155: how faithful is the liaison?. Biochim Biophys Acta.

[20] Kang SJ, Lee HM, Park YI, Yi H, Lee H, So B (2016). Chemically induced hepatotoxicity in human stem cell-induced hepatocytes compared with primary hepatocytes and HepG2. Cell Biol Toxicol.

[21] Kang SJ, Park YI, Kwon MJ, Yang YH, Bang SI, Sohn SH, Park YH, So B, Kang HG (2015). Adipose stromal cells are a more efficient source than adipose stem cells in retrovirus-mediated iPS induction. Cell Mol Bioeng.

[22] Bieberich E (2008). Smart drugs for smarter stem cells: making SENSe (sphingolipid-enhanced neural stem cells) of ceramide. Neurosignals.

[23] DeLaForest A, Nagaoka M, Si-Tayeb K, Noto FK, Konopka G, Battle MA (2011). HNF4A is essential for specification of hepatic progenitors from human pluripotent stem cells. Development.

[24] Hay DC, Fletcher J, Payne C, Terrace JD, Gallagher RC, Snoeys J (2008). Highly efficient differentiation of hESCs to functional hepatic endoderm requires ActivinA and Wnt3a signaling. Proc Natl Acad Sci U S A.

[25] Ogawa S, Surapisitchat J, Virtanen C, Ogawa M, Niapour M, Sugamori KS (2013). Three-dimensional culture and cAMP signaling promote the maturation of human pluripotent stem cell-derived hepatocytes. Development.

[26] Godoy P, Hewitt NJ, Albrecht U, Andersen ME, Ansari N, Bhattacharya S (2013). Recent advances in 2D and 3D in vitro systems using primary hepatocytes, alternative hepatocyte sources and non-parenchymal liver cells and their use in investigating mechanisms of hepatotoxicity, cell signaling and ADME. Arch Toxicol.

[27] Lee AS, Tang C, Cao F, Xie X, van der Bogt K, Hwang A (2009). Effects of cell number on teratoma formation by human embryonic stem cells. Cell Cycle.

