# Supplementary material for: Hepatic population derived from human pluripotent stem cells is effectively increased by selective removal of undifferentiated stem cells using YM155
Source: Stem Cell Res Ther. 2017 Apr 17;8:78. doi: 10.1186/s13287-017-0517-2 (PMC5392904; doi:10.1186/s13287-017-0517-2)
Supplement: Supplementary file 1 — is Table S1. presenting a list of primer sets and Table S2. presenting antibody information. (DOCX 31 kb) [file 13287_2017_517_MOESM1_ESM.docx]

**Supplementary data**

Table S1. List of primer sets

| Primer sets | | Sequences (5’-3’) | Temp.  (°C) | Accession # |
| --- | --- | --- | --- | --- |
|  |  |  |  | References |
| *NANOG* | F | TGAACCTCAGCTACAAACAG | 53 | NM_024865.2 |
|  | R | TGGTGGTAGGAAGAGTAAAG |  |  |
| *OCT4* | F | GACAGGGGGAGGGGAGGAGCTAGG | 58 | NM_002701.4 |
|  | R | CTTCCCTCCAACCAGTTGCCCCAAA |  |  |
| *CXCR4* | F | CACCGCATCTGGAGAACCA | 55 | NM_003467 |
|  | R | GCCCATTTCCTCGGTGTAGTT |  |  |
| *SOX17* | F | CCAGAATCCAGACCTGCACAA | 57 | NM_022454.3 |
|  | R | CTCTGCCTCCTCCACGAA |  |  |
| *GATA4* | F | CATCAAGACGGAGCCTGGCC | 60 | NM_002052 |
|  | R | TGACTGTCGGCCAAGACCAG |  |  |
| *FOXA2* | F | AGATGGAAGGGCACGAGC | 56 | NM_153675.2 |
|  | R | CAGGCCGGCGTTCATGTT |  |  |
| *ALB* | F | GGTGTTGATTGCCTTTGCTC | 56 | NM_000477 |
|  | R | CCCTTCATCCCGAAGTTCAT |  |  |
| *HNF4A* | F | CCACGGGCAAACACTACGG | 56 | NM_000457 |
|  | R | GGCAGGCTGCTGTCCTCAT |  |  |
| *AFP* | F | TTTTGGGACCCGAACTTTCC | 56 | NM_001134.1 |
|  | R | CTCCTGGTATCCTTTAGCAACTCT |  |  |
|  |  |  |  |  |
|  |  |  |  |  |
| *PAX6* | F | TGTCCAACGGATGTGTGAGT | 60 | NM_001604.4 |
|  | R | TTTCCCAAGCAAAGATGGAC |  |  |
| *NESTIN* | F | GAAACAGCCATAGAGGGCAAA | 60 | NM_006617 |
|  | R | TGGTTTTCCAGAGTCTTCAGTGA |  |  |
| *NCAM* | F | GTGCTGTCCAACAACTAC | 55 | NM_024003 |
|  | R | AATGACCTGAATATCTTTGAAGT |  |  |
| *BRACHYURY T* | F | ACCCAGTTCATAGCGGTGAC | 56 | NM_003181.2 |
|  | R | CCATTGGGAGTACCCAGGTT |  |  |
| *IGF2* | F | GAAACAATTGGCAAAATAAAGG | 60 | NM_000612 |
|  | R | CCAGTTTACCCTGAAAATTCC |  |  |
| *MSX1* | F | CGAGAGGACCCCGTGGATGCAGAG | 55 | NM_002448.3 |
|  | R | GGCGGCCATCTTCAGCTTCTCCAG |  |  |
| *TNNT2*  *(TnTc)* | F | ATGAGCGGGAGAAGGAGCGGCAGAAC | 55 | NM_00100143 2.1 |
|  | R | TCAATGGCCAGCACCTTCCTCCTCTC |  |  |
| *BIRC5*  *(Survivin)* | F | GGACCACCGCATCTCTACAT | 56 | NM_001012270 |
|  | R | GCACTTTCTTCGCAGTTTCC |  |  |
| *BCL-2* | F | TCCATGTCTTTGGACAACCA | 56 | NM_000633 |
|  | R | CTCCACCAGTGTTCCCATCT |  |  |
| *BAX* | F | TCTGACGGCAACTTCAACTG | 56 | NM_001291428 |
|  | R | TTGAGGAGTCTCACCCAACC |  |  |
| *GAPDH* | F | CAAAGTTGTCATGGATGACC |  | NM_002046 |
|  | R | CCATGGAGAAGGCTGGGG |  |  |

Table S2. Information of antibodies.

| Primary antibody | |  | Secondary antibody | |
| --- | --- | --- | --- | --- |
| Antibodies | Manufacture  (Cat. #) |  | Antibodies | Manufacture  (Cat. #) |
| Anti-NANOG | Stemgent (09-0020) |  | Goat anti-rabbit IgG-Cy3 | Stemgent (09-0037) |
| Anti-OCT4 | Stemgent (09-0023) |  | Goat anti-rabbit IgG-Cy3 | Stemgent (09-0037) |
| Anti-SSEA-4 | Stemgent (09-0003) |  | Conjugated to PE | - |
| Anti-TRA-1-60 | Stemgent (09-0068) |  | Conjugated to FITC | - |
| Anti-CXCR4 | Abcam (ab2074) |  | Goat anti-rabbit IgG-Cy3 | Stemgent (09-0037) |
| Anti-SOX17 | Millipore (09-038) |  | Goat anti-rabbit IgG-Cy3 | Stemgent (09-0037) |
| Anti-GATA | Santa Cruz Biotech  (sc-1237) |  | Donkey anti-goat IgG (H+L)-FITC | Invitrogen (A11055) |
| Anti-CK18 | Santa Cruz Biotech  (sc-28264) |  | Goat anti-rabbit IgG-Cy3 | Stemgent (09-0037) |
| Anti-ALB | Abcam (ab131176) |  | Goat anti-mouse IgM+IgG-Cy3 | Stemgent (09-0036) |
| Anti-AFP | Santa Cruz Biotech  (sc-8108) |  | Donkey anti-goat IgG (H+L)-FITC | Invitrogen (A11055) |
| Anti-CYP1A2 | Santa Cruz Biotech  (sc-53614) |  | Goat anti-mouse IgM+IgG-Cy3 | Stemgent (09-0036) |
| Anti-CYP3A4 | Santa Cruz Biotech  (sc-53850) |  | Goat anti-mouse IgM+IgG-Cy3 | Stemgent (09-0036) |

All of primary and secondary antibodies were diluted as 1:100.
