# Supplementary material for: Hepatic population derived from human pluripotent stem cells is effectively increased by selective removal of undifferentiated stem cells using YM155
Source: Stem Cell Res Ther. 2017 Apr 17;8:78. doi: 10.1186/s13287-017-0517-2 (PMC5392904; doi:10.1186/s13287-017-0517-2)

**Additional File 2; Supplementary Figures**

**Materials and Methods**

**TRA-1-60 livestaining**

To conduct livestaining of TRA-1-60, the differentiating cells were washed with 1X PBS after the supernatants were removed. StainAlive TRA-1-60 (DyLight488, Stemgent) was diluted with Hep-1 medium at a ratio of 1:100. The medium was added, and the cells were incubated for 30 min at 37°C. The fluorescence was observed under a fluorescence microscope (Axiovert).

**Annexin V staining**

To conduct staining of Annexin V, we used PE-Annexin V Apoptosis detection kit I (BD Bioscience). QIA7, QIA7-iHeps and hAT-SCs were treated by serial concentrations of YM155 (1–100 nM) for 24 h. After the supernatants were removed, Annexin V was diluted with mTeSR1, Hep-1 and hAT-SC medium at a ratio of 1:100. Each medium was added, and the cells were incubated for 10 min at room temperature. The fluorescence was observed under a fluorescence microscope (Axiovert).

Figure S1. Immunocytochemical phenotype stage on the hepatic differentiation. QIA7 was differentiated to hepatocytes and the expressions of marker proteins were evaluated at the final day of each stage.


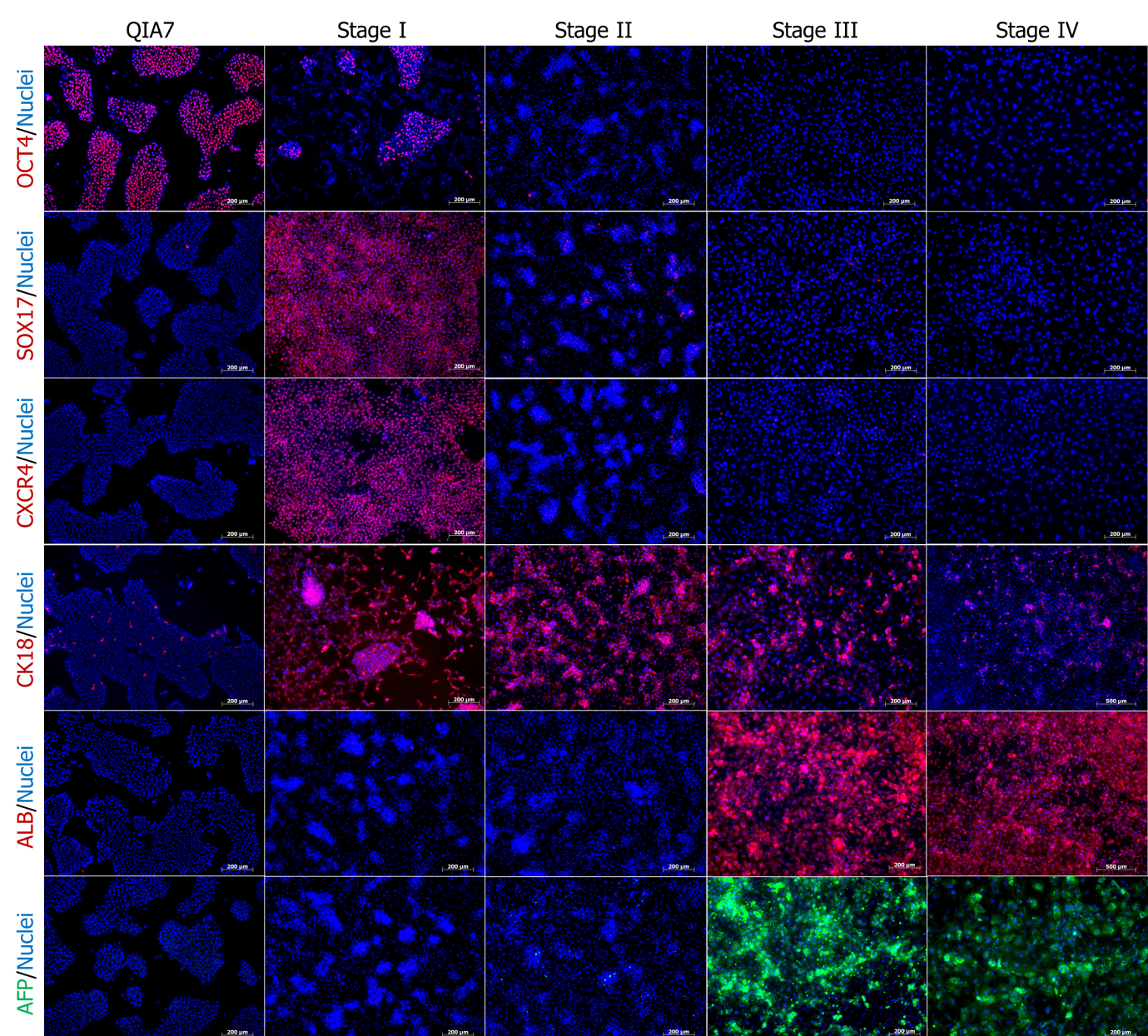


Figure S2. YM155 effect on Stage I. Most of QIA7 and differentiating cells were dead after YM155 treatment (10 nM) at Stage I compared to control (DMSO).


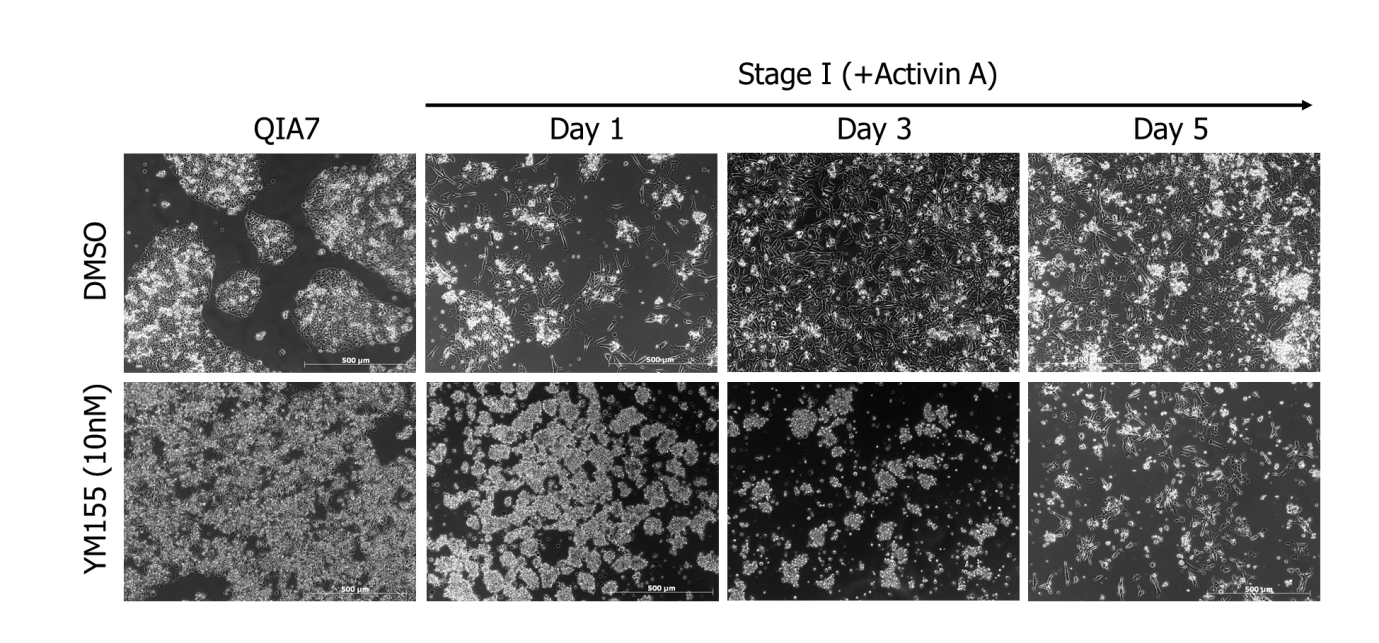


Figure S3. The expressions of apoptosis-related genes (*BIRC5* and *BAX*) during the whole hepatic differentiation of QIA7.


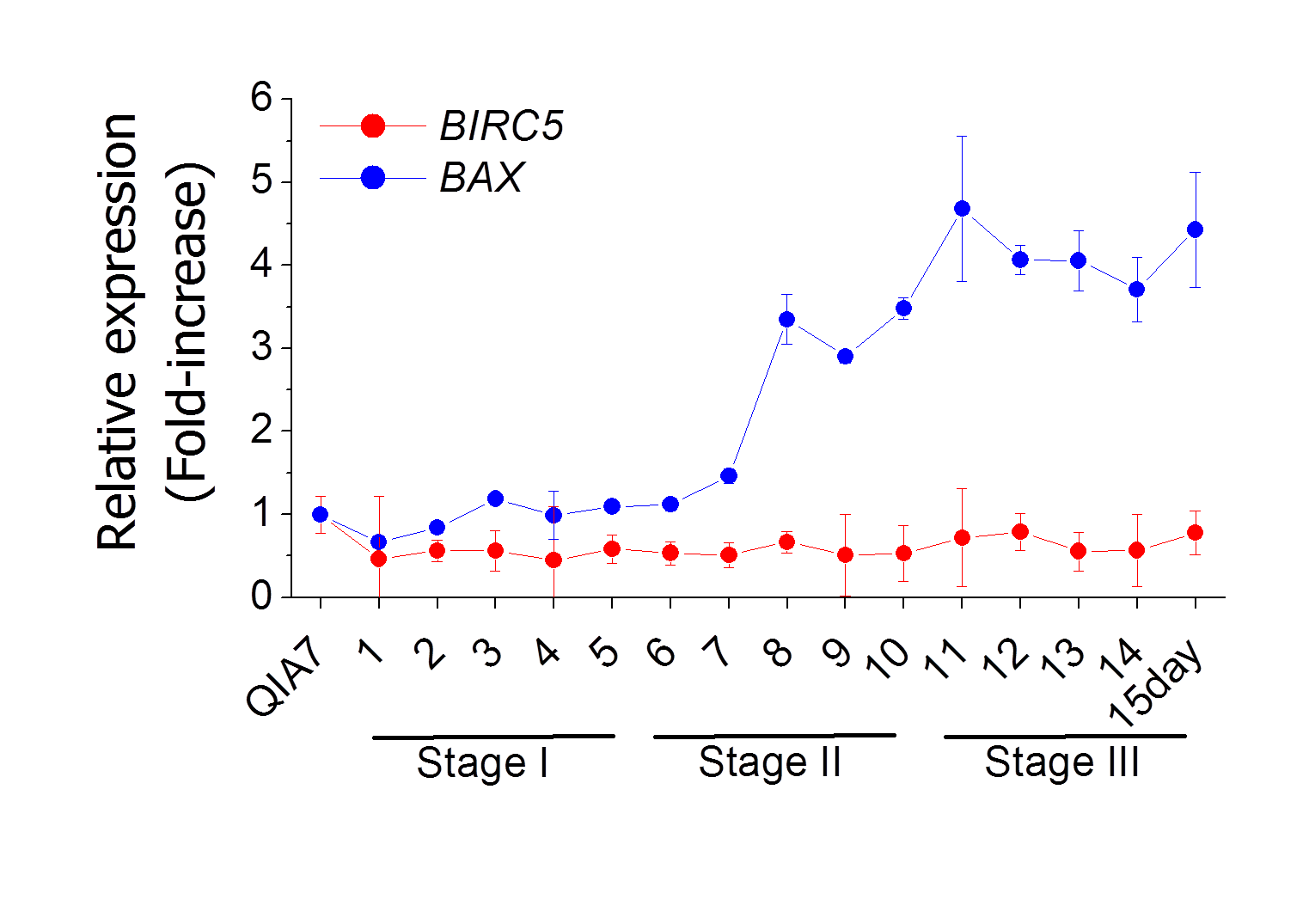


Figure S4. Annexin V staining with QIA7, QIA7-iHeps (7 days of differentiation) and hAT-SCs after YM155 treatment for 24 h.


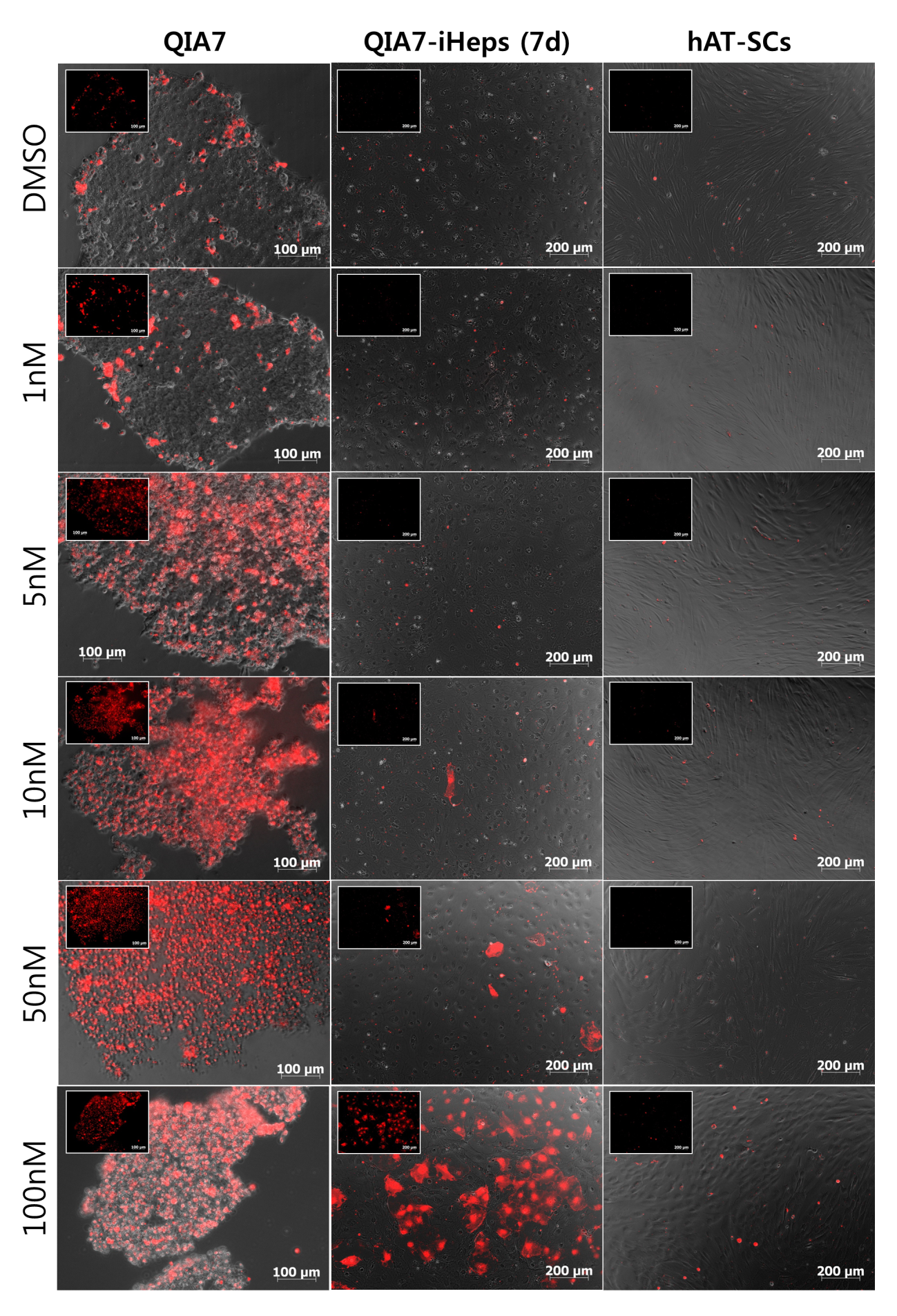


Figure S5. Livestaining of TRA-1-60 at Stage II (day 7 of differentiation) after YM155 treatment for 24 h.


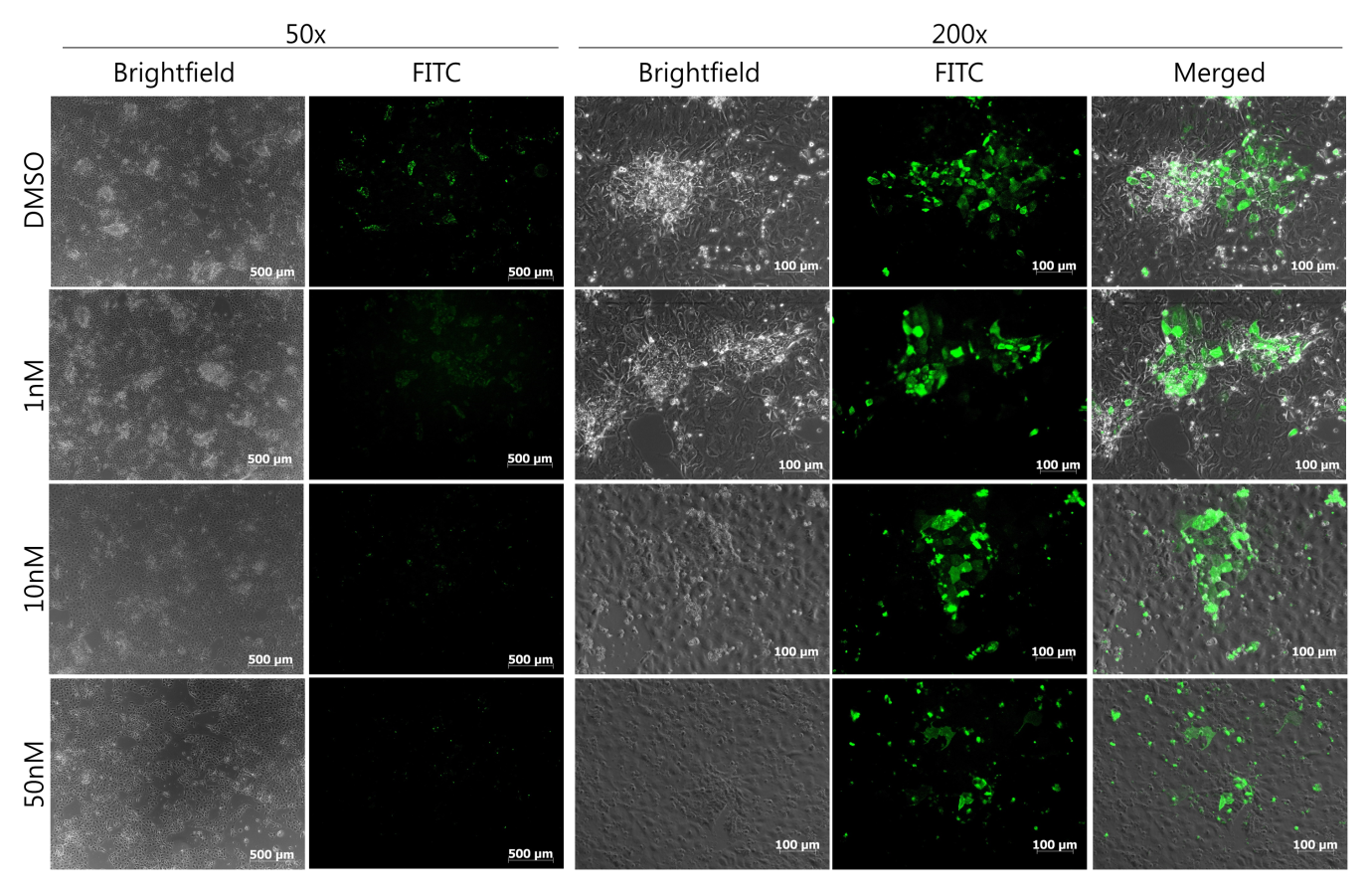


Figure S6. Histological analysis of teratomas derived from QIA7 and QIA7-iHeps without pretreatment of YM155.


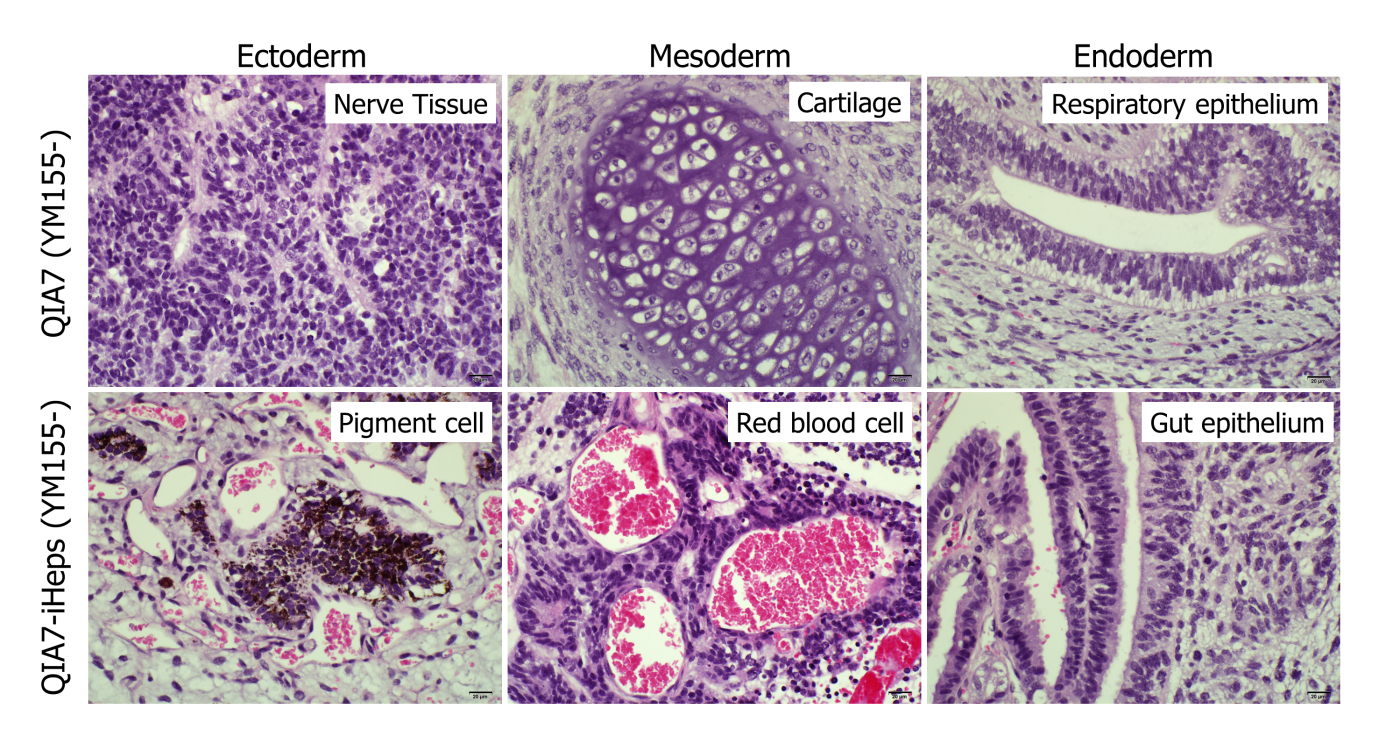

Supplement: Supplementary file 2 — contains supplementary materials and methods presenting TRA-1-60 livestaining and Annexin V staining; Figure S1. showing the immunocytochemical phenotype stage on hepatic differentiation; Figure S2. showing the YM155 effect on stage I; Figure S3. showing expression of apoptosis-related genes (BIRC5 and BAX) during the whole hepatic differentiation of QIA7; Figure S4. showing Annexin V staining with QIA7, QIA7-iHeps (7 days of differentiation) and hAT-SCs after YM155 treatment for 24 h; Figure S5. showing the livestaining of TRA-1-60 at stage II (day 7 of differentiation) after YM155 treatment for 24 h; and Figure S6. showing the histological analysis of teratomas derived from QIA7 and QIA7-iHeps without pretreatment of YM155. (DOCX 11505 kb) [file 13287_2017_517_MOESM2_ESM.docx]
